# 

**Published:** 1914-01-17

**Authors:** 


### A Doctor's Bequests.

Mr. Edward Nettleship, F.R.C.S., of Hindhead, Surrey, who died on October 30, left estate sworn at £26,768 gross, and he bequeathed, as payable after his wife's death, £3,000 to St. Thomas's Hospital for the creation or augmentation of a fund for the endowment of lectureships in the medical school, and £1,000 to the Royal London Ophthalmic Hospital. He also left a share of his residuary estate after the death of his wife as follows:—If the ultimate residue of his estate exceeds £9,000, then £2,000 to the medical school of St. Thomas's Hospital for the same purposes as the above-mentioned legacy to them, and £1,000 to the Royal London Ophthalmic Hospital. If the ultimate residue is less than £9,000 but over £6,000 the share of the hospitals is to be reduced accordingly; but if it is under £6,000 the whole is given to relations of the testator.

RATES OF SUBSCRIPTION: Twelvemonths, 6 6; Six months, 4/-; Three months, 2/-, post free to any address in the United Kingdom. Abroad, Twelvemonths, 8/8; Six months, 5/-; Three months, 2/6, post free.—Address The Subscription Dept., "THE HOSPITAL," The Hospital Building, 8/292 Southampton Street, Strand, London, W.C.

